# Supplementary material for: Confirmation of Large Language Models in Head and Neck Cancer Staging
Source: Diagnostics (Basel). 2025 Sep 18;15(18):2375. doi: 10.3390/diagnostics15182375 (PMC12468830; doi:10.3390/diagnostics15182375)
Supplement: Supplementary file 1 [file diagnostics-15-02375-s001.zip › diagnostics-3839298-supplementary.pdf]

Supplementary Table S1. Clinician-Performed Staging

Cancer localization	**T** N(%)	**N** N(%)	**M** N(%)	**Stage** N(%)
**Overall**	T0 1 (0.5)<br>T1 18 (8.9)<br>T2 49 (24.3)<br>T3 64 (31.7)<br>T4 70 (34.6)	N0 73 (36.1)<br>N1 34 (16.8)<br>N2 61 (30.1)<br>N3 31 (15.3)	M0 202 (100)<br>M1 0 (0)	1 11 (5.4)<br>2 23 (11.4)<br>3 58 (28.7)<br>4 110 (54.4)
**Larynx**	T1a 10 (10.9)<br>T1b 0 (0)<br>T2 16 (17.4)<br>T3 36 (39.1)<br>T4a 30 (32.6)<br>T4b 0 (0)	N0 46 (50)<br>N1 10 (10.9)<br>N2a 4 (4.3)<br>N2b 8 (8.7)<br>N2c 10 10 10.9)<br>N3a 0 (0)<br>N3b 14 (15.2)	M0 92 (100)<br>M1 0 (0)	1 9 (9.8)<br>2 12 (13)<br>3 21 (22.8)<br>4A 36 (39.1)<br>4B 14 (15.2)<br>4C 0(0)
**Hypopharynx**	T1 0 (0)<br>T2 1 (7.7)<br>T3 4 (30.8)<br>T4a 8 (61.5)<br>T4b 0(0)	N0 4 (30.8)<br>N1 2 (15.4)<br>N2a 1 (7.7)<br>N2b 1 (7.7)<br>N2c 3 (23.1)<br>N3a 0 (0)<br>N3b 2 (15.4)	M0 13 (100)<br>M1 0 (0)	1 0 (0)<br>2 1 (7.7)<br>3 2 (15.4)<br>4A 8 (61.5)<br>4B 2 (15.4)<br>4C 0(0)
**Oral cavity/ Oropharynx**	T1 4 (8.7)<br>T2 15 (32.6)<br>T3 13 (28.3)<br>T4a 12 (26.1)<br>T4b 2 (4.3)	N0 13(28.3)<br>N1 14 (30.4)<br>N2a 4 (8.7)<br>N2b 4(8.7)<br>N2c 3(6.5)<br>N3a 0(0)<br>N3b 8(17.4)	M0 46 (100)<br>M1 0 (0)	1 1 (2.2)<br>2 6 (13)<br>3 14 (30.4)<br>4A 15 (32.6)<br>4B 10 (21.7)<br>4C 0(0)
**Nasopharynx**	T0 1 (2.3)<br>T1 4(9.3)<br>T2 15 (32.6)<br>T3 11 (25.6)<br>T4 12 (27.9)	N0 3 (7)<br>N1 8 (18.6)<br>N2 25 (58.1)<br>N3 7 (16.3)	M0 43 (100)<br>M1 0 (0)	1 1 (2.3)<br>2 2 (4.7)<br>3 21 (48.8)<br>4A 19 (44.2)<br>4B 0(0)
**Salivary**	T0 0 (0)<br>T1 0 (0)<br>T2 1 (100)<br>T3 0 (0)<br>T4 0 (0)	N0 1(100)<br>N1 0<br>N2 0<br>N3 0	M0 1 (100)<br>M1 0 (0)	1 0 (0)<br>2 1(100)<br>3 0(0)<br>4A 0(0)<br>4B 0(0)<br>4C 0(0)
**Nasal cavity and sinuses**	T1 0 (0)<br>T2 1 (14.3)<br>T3 0 (0)<br>T4a 5(71.4)<br>T4b 1(14.3)	N0 6(85.7)<br>N1 0 (0)<br>N2 1 (14.3)<br>N3 0 (0)	M0 1 (100)<br>M1 0 (0)	1 0(0)<br>2 1(14.3)<br>3 0(0)<br>4A 5(71.4)<br>4B 1(14.3)<br>4C 0(0)

Supplementary Table S2.TNM Stage Accordancy Between LLM and Clinician

LLM	**T** K(sd)	**Pt** K(sd)	**N** K(sd)	**Pn** K(sd)	**Stage** K(sd)	**P**	**M**
**ChatGPT**	0.706(0.04)	pt1 0.033<br>pt2 0.419<br>pt3 0.002	0.823(0.03)	pn1<0.001<br>pn2 0.004<br>pn3 0.034	0.797(0.03)	p1 <0.001<br>p2= 0.071<br>p3= 0.021	-
**DeepSeek**	0.577 (0.04)		0.561(0.03)		0.522(0.04)		-
**Grok**	0.526 (0.04)		0.720(0.03)		0.614(0.04)		-

P1 ChatGPT stage vs. DeepSeek stage, p2 DeepSeek stage vs. Grok stage p3 ChatGPT stage vs. Grokstage

pt1 ChatGPTt vs. DeepSeekt, pt2 DeepSeekt vs. Grokt pt3 ChatGPTt vs. Grokt

pn1 ChatGPTn vs. DeepSeekn, pn2 DeepSeekn vs. Grokn pn3 ChatGPTn vs. Grokn

Abbreviations: LLM = Large Language Model, SD = standart deviation

Bonferroni correction (α = 0.05/3 = 0.0167).

Supplementary Table S3.Concordance Rates of T, N, and Stage According to Clinical and Pathological Staging

	**ChatGPT**	**DeepSeek**	**Grok**
Clinical Staging<br>Pathological Staging	104(85.2) k0.781<br>69 (86.3) k0.811<br>p0.67	88(72.1) k 0.574<br>48 (60) k 0.444<br>p 0.18	90(73.8) K 0.571<br>62 (77.2) K 0.661<br>p 0.311
Clinical T Staging<br>Pathological T Staging	96(78.7) k0.710<br>63(78.8) k 0.699<br>p 0.894	85 (69.7) k 0.593<br>54(67.5) k 0.553<br>P 0.687	75 (61.5) k 0.513<br>55(68.8) k 0.542<br>p 0.750
Clinical N Staging<br>Pathological N Staging	105 (86.1) k 0.800<br>71 (88.1) k 0.851<br>p 0.418	82 (67.2) k 0.564<br>53 (66.3) k 0.549<br>p 0.870	95(77.9) k 0.680<br>67 (83.8) k 0.768<br>p 0.244

Supplementary Table S4. Concordance Rates by Staging Methods

		**Only Imaging n(%)**	**Only Pathology n(%)**	**Only Examinatinon n(%)**	**Combined n(%)**	**p**
**ChatGPT**	**T**	40(81.46)	2(66.7)	1(100)	116(77.9)	0.683
	**N**	38(77.6)	3(100)	1(100)	134(89.9)	0.158
	**Stage**	38 (77.6)	3(100)	1(100)	131(87.9)	0.140
**DeepSeek**	**T**	34(69.4)	2(66.7)	1(100)	139(68.8)	1
	**N**	30(61.2)	2(66.7)	1(100)	102(68.5)	0.762
	**Stage**	30(61.2)	2(66.7)	1(100)	103(69.1)	0.733
**Grok**	**T**	28(57.1)	2(66.7)	0(0)	100(67.1)	0.270
	**N**	35(71.4)	3(100)	1(100)	123(82.6)	0.319
	**Stage**	37(75.5)	2(66.7)	1(100)	112(75.2)	1

Supplementary Table S5 .F1 Scores and 95% Confidence Intervals by Model and Stage

Model	T<br>F1 Score<br>(%95 CI)	N<br>F1Score<br>(%95 CI)	M<br>F1Score<br>(%95 CI)	S<br>F1Score<br>(%95 CI)
**ChatGPT**	0.78(0.72-0.83)	0.86(0.80-0.90)	-	0.85(0.80-0.89)
**DeepSeek**	0.69(0.63-0.75)	0.66(0.58-0.71)	-	0.65(0.60-0.73)
**Grok**	0.64(0.58-0.71)	0.78(0.72-0.83)	-	0.72(0.65-0.77)

Supplementary Table S6. Concordance rate by gender

Staging	Concordance Rate (%) Female	Concordance Rate (%) Male	p-value
**ChatGPT**			
T	78.4	78.8	0.955
N	86.3	87.4	0.833
M	100	100	1
Stage	90.2	84.1	0.284
**DeepSeek**			
T	66.7	69.5	0.702
N	58.8	69.5	0.16
M	100	100	1
Stage	70.6	66.2	0.566
**Grok**			
ST	58.8	66.2	0.340
N	72.5	82.8	0.113
M	100	100	1
Stage	74.5	75.5	0.888

Supplementary Table S7. Concordance Rates by Surgical Status

Staging<br>S	Accuracy Rate (%)Definitive Surgery Performed	Accuracy Rate (%)Definitive Surgery Not Performed	p-value
**ChatGPT**			
T	78	79.2	0.849
N	90.2	85.0	0.274
M	100	100	1
Stage	86.6	85.0	0.752
**DeepSeek**			
T	67.1	70.0	0.659
N	68.3	65.8	0.715

M	100	100	1
Stage	62.2	70.8	0.199
**Grok**			
T	69.5	60.8	0.206
N	85.4	76.7	0.128
M	100	100	1
Stage	78.0	73.4	0.446

Supplementary Table S8. Concordance Rates by TNM Staging

**ChatGPT**					
**Variable**	**Subgroup**	**Correct (n)**	**Total (n)**	**Rate (%)**	**p**
**T**	T0	1	1	100.0%	**0.009**
	T1	13	18	72.2%	
	T2	34	49	69.4%	
	T3	46	63	73.0%	
	T4	65	71	91.5%	
**N**	N0	70	73	95.9%	**0.021**
	N1	28	35	80.0%	
	N2	51	62	82.3%	
	N3	27	32	84.4%	
**M**	M0	202	202	100%	**1**
	M1	0	0	0	
**Stage**	Stage 1	8	11	72.7%	**0.021**
	Stage 2	15	23	65.2%	
	Stage 3	51	58	87.9%	
	Stage 4A	76	83	91.6%	
	Stage 4B	23	27	85.2%	
**DeepSeek**					
**T**	T0	1	1	100.0%	0.018
	T1	10	18	55.6%	

	T2	28	49	57.1%	
	T3	42	63	66.7%	
	T4	58	71	81.7%	
**N**	N0	66	73	90.4%	0.000
	N1	29	35	82.9%	
	N2	28	62	45.2%	
	N3	12	32	37.5%	
**M**	M0	202	202	100%	1
	M1	0	0	0	
**Stage**	Stage 1	6	11	54.5%	0.000
	Stage 2	15	23	65.2%	
	Stage 3	42	58	72.4%	
	Stage 4A	66	83	79.5%	
	Stage 4B	7	27	25.9%	
**Grok**					
**T**	T0	1	1	100.0%	0.000
	T1	14	18	77.8%	
	T2	20	49	40.8%	
	T3	35	63	55.6%	
	T4	60	71	84.5%	
**N**	N0	69	73	94.5%	0.000
	N1	27	35	77.1%	
	N2	45	62	72.6%	
	N3	21	32	65.6%	
**M**	M0	202	202	100%	0
	M1	0	0	0	
**Stage**	Stage 1	7	11	63.6%	0.147
	Stage 2	14	23	60.9%	
	Stage 3	42	58	72.4%	
	Stage 4A	69	83	83.1%	
	Stage 4B	20	27	74.1%	

Supplementary Figure S1. Example of an interaction with a Large Language Model (LLM), illustrating the application of a prompt for TNM staging.

Boyun BT: Krikoid posterior başlangıcı düzeyinde yaklaşık 5 mm boyutunda kalsifikasyon, bu düzeyde özofagus mukozasında asimetrik minimal kalınlaşma (travmaya sekonder) ve ayrıca bilateral aryeepiglottik fold düzeyinde hipofarinkste posteriorda yaklaşık 6 mm kalınlığa ulaşan asimetrik mukozal kalınlaşma izlenmiş. Patolojik boyutta boyun lenf nodu saptanmamış
Boyun MRG : Hipofarenkste özofagus başlangıç kesiminde yaklaşık 10x28 mm boyutunda hipofarenks lümenine doğru polipoid uzanım gösteren ve her iki periepiglottik vold komşuluğuna uzanım gösteren T2A ve STIRAda hiperintens T1Ada kas ile izointens IVKM sonrasında heterojen kontrastlanma gösteren yumuşak doku kitlesi izlenmiş.
PET-BT : MRG'de hipofarenkste tanımlanan yumuşak doku yapılanmasında yoğun patolojik 18F-FDG tutulumuna (SUVmax: 20.7) ek olarak boyun her iki yanında juguler zincirde mevcut lenf nodlarında patolojik 18F-FDG tutulumu (SUVmax: 8.3) gözlenmiş. hipofarenks arka duvardan alınan biyopsi sonucu " Yassı Hücreli Karsinom" TNM EVRESİ NEDİR

Hastanızın boyun BT, MRG ve PET-BT bulguları ve biyopsi sonucu göz önünde bulundurulduğunda TNM evresi aşağıdaki gibi değerlendirilebilir:

🧠 Klinik Bilgilerin Özeti

- **BT:** Hipofarinks ve özofagus mukozasında asimetrik kalınlaşma.
- **MRG:** Hipofarinks lümenine doğru polipoid uzanım gösteren, periepiglottik volda yerleşimli 10×28 mm'lik kitle.
- **PET-BT:**
  - Kitlede **SUVmax: 20.7**
  - **Her iki juguler zincirde** patolojik lenf nodu tutulumu (SUVmax: 8.3)
- **Patoloji:** **Yassı hücreli karsinom**
- **Uzak metastaz:** Yok

🩺 TNM Evrelemesi (AJCC 8. baskıya göre)

✅ **T evresi (T3)**

- MRG'deki kitlenin boyutu ve hipofarinks lümenine uzanımı, periepiglottik boşluklara komşuluğu dikkate alındığında:
  - **T3:** Tümör, hipofarinkste 4 cm'yi aşmasa da periepiglottik boşluğa uzanıyor (veya tek başına kitlenin boyutu >4 cm olmasa da derin invazyon düşündürüyor).

✅ **N evresi (N2c)**

- **Her iki juguler zincirde** lenf nodu tutulumu mevcut:
  - **N2c: Kontralateral ya da bilateral lenf nodu tutulumu**, her biri ≤6 cm.

✅ **M evresi (M0)**

- Uzak metastaz bildirilmemiş.

🔎 Sonuç: Klinik TNM Evresi

cT3 N2c M0

(= **Evre IVA** – AJCC 8. baskıya göre hipofarinks kanserlerinde)

14.04.2022 tarihli Boyun bt: Epiglot düzeyinde sağda belirgin her iki vallekulayı oblitere eden, glossoepiglottik katlantıyı tutan, preepiglottik boşluğu dolduran, sol pirifom sinüse uzanan aksiyal planda en geniş yerinde 3.5x2 cm ölçülen heterojen kontrastlanan kitlesel yumuşak doku izlenmektedir. Dil kökü ile yer yer ara yağ planları net seçilememektedir. Sol aryoepiglottik katlantı da asimetrik kalındır (infiltre?). Kitlenin Malign olduğu düşünülmektedir. Histopatolojik değerlendirme önerilir. Her iki servikal zincirde patolojik boyutlara ulaşmayan büyüğünün kısa boyutu 8 mm ölçülen lenf bezleri izlenmektedir.
KBB muuayenesi: Bilateral CV hareketli, glottik açıklık yeterli izlendi. Epiglot ödemli kitlesel lezyon izlendi. Sol bant ventrikülde ve epiglot anterıor yüzünde ülserovejetan kitle izlendi.
14,4,22 pet: Epiglottis lojunda izlenen lobule konturlu kitlede yoğun patolojik 18f-FDG tutulumu (SUVmax.13.2) izlenmiştir. Bilateral servikal, juguler zincirde mevcut milimetrik lenf nodlarında geç görüntülerde belirgin artış göstermeyen hafif artmış 18F-FDG tutulumu (SUVmax:2.5) gözlenmiştir (reaktif ?, hastanın primer patoloji nedeniyle klinik korelasyonu önerilir). Sağ akciğer alt lob posteriobazalde mevcut milimetrik nodülde (SUVmax:1.4) ve bilateral hiler-bronkopulmoner bölgede (SUVmax.2.3) artmış 18F-FDG tutulumları mevcuttur (benign patolojiler ?). Vücutta diğer alanlarda patolojik 18F-FDG tutulumu saptanmamıştır.
13/04/2022 LARİNGOSKOPİ+BİYOPSİ ; Patoloji:yassı hücreli karsinom, epiglot ve bant ventrikül örnekleri
TNM EVRESİ NEDİR

TNM Evresi:

- **T3 N0 M0**

Evre Grubu (AJCC 8. Baskı):

Supraglottik larenks kanserinde:

- **Evre I:** T1 N0 M0
- **Evre II:** T2 N0 M0
- **Evre III:** T3 N0 M0 veya T1-T3 N1 M0
- **Evre IVA:** T4a N0-N2 M0 veya T1-T4a N2 M0
- **Evre IVB:** T4b herhangi N M0 veya herhangi T N3 M0
- **Evre IVC:** Herhangi T, herhangi N, M1

Sonuç: T3 N0 M0 → **Evre III**

Nihai Cevap:

Bu hastanın TNM evresi, mevcut verilere göre **T3 N0 M0**'dur ve bu da **Evre III** supraglottik larenks kanserine karşılık gelir. Ancak dil kökü invazyonu veya lenf nodu metastazı histopatolojik olarak doğrulanırsa evre T4a veya N2c olarak güncellenebilir. Bu durumda klinik ve patolojik korelasyon önemlidir.
